# The Immune Interplay between Thyroid Papillary Carcinoma and Hepatic Fibrosis

**DOI:** 10.1371/journal.pone.0132463

**Published:** 2015-07-07

**Authors:** Nidal Muhanna, Johnny Amer, Ahmad Salhab, Jean-Yves Sichel, Rifaat Safadi

**Affiliations:** 1 Liver and Gastroenterology Units, Division of Medicine, Hadassah University Medical Center, Jerusalem, Israel; 2 Department of Otolaryngology, Head & Neck Surgery, Shaare Zedek Medical Center, Jerusalem, Israel; Bambino Gesu' Children Hospital, ITALY

## Abstract

**Background:**

A high prevalence of thyroid papillary cancer was reported in hepatitis-C-virus (HCV) positive patients. However, the mechanistic role of hepatic-fibrosis in thyroid malignancy progressions is still unclear.

**Aim:**

We aimed to study the immune-modulatory interactions between thyroid papillary carcinoma and hepatic-fibrosis.

**Methods:**

Hepatic-fibrosis was induced in nude-nu-male mice by intra-peritoneal administration of carbon-tetrachloride. To induce thyroid-tumor, a thyroid papillary carcinoma cell line (NPA) was injected subcutaneously in the backs. Fibrotic profile was estimated by α-smooth-muscle-actin (αSMA) expression in liver tissue extracts using western-blots and RT-PCR. Intra-hepatic NK cells were isolated and stained for NK activity (CD107a) by flow cytometry. Liver histopathology (H&E staining), thyroid tumor mass and serum alanine aminotransferase (ALT), serum vascular endothelial growth factor (VEGF) and free-T4 levels were also assessed.

**Results:**

*Ex-vivo*: NPA cells were co-cultured with intra-hepatic NK cells isolated from fibrotic mice with/without the tumor were analyzed for CFSE-proliferations. Both tumor groups (with/without hepatic-fibrosis) excreted higher serum free T4 levels. Hepatic-fibrosis increased tumor weight and size and serum free-T4 levels. In addition, tumor induction increased liver injury (both hepatic-fibrosis, necro-inflammation and serum ALT levels). In addition, tumor-bearing animals with hepatic-fibrosis had increased NK activity. NPA tumor-bearing animals increased fibrosis in spite of increased NK activity; probably due to a direct effect through increased serum free-T4 excretions. Serum VEGF levels were significantly increased in the fibrotic- bearing tumor groups compared to the non-fibrotic groups. *In-vitro*, NK cells from fibrotic tumor-bearing animals reduced proliferation of NPA cells. This decrease is attributed to increase NK cells activity in the fibrotic animals with the NPA tumors.

**Conclusions:**

Our results propose that NK cells although were stimulated in advanced fibrosis with tumor, they lost their anti-tumor and anti-fibrotic activity probably due to secretions of T4 and VEFG and may explain increased risk of thyroid tumors in chronic HCV patients.

## Introduction

Thyroid cancer is the most common endocrine malignancy and accounts for the majority of endocrine cancer deaths each year [1, 2]. Papillary subtype of thyroid carcinoma (PTC) is the most common of all thyroid cancers and represents more than 75% of thyroid cancer [3]. A rise in thyroid cancer incidence, especially of the papillary type, has been reported in several countries, including the United States, during the past several decades, and the factors responsible for the increase remain unknown [3]. Overall, thyroid cancer is three times more common in women than men, with the greatest gender differences observed between the ages of 25 and 64 [4].The striking gender differences in incidence strongly suggest that sex steroid hormones may be involved in the development of this disease [5]. On the other hand, it is well known that clinical signs of hypogonadism are common in patients with liver fibrosis [6].

In addition to hepatic-fibrosis, an oncogenic role of hepatitis C virus (HCV) in the pathogenesis of different kinds of extra-hepatic tumors has been suggested; for example, malignant lymphoma and pancreatic cancer [7, 8]. Furthermore, some studies have reported a high prevalence of thyroid papillary cancer in patients with HCV infection [9–13]. Exactly what mechanisms transduce the HCV oncogenic potential in thyroid cancer remains to be investigated.

Hepatic fibrosis is the result of chronic liver injury, regardless of etiology, during which hepatic stellate cells (HSCs) proliferate and differentiate into matrix-producing cells [14]. HSCs represent main source of scar tissue during liver fibrosis and cirrhosis [15, 16]. Our previous studies showed that CD8 lymphocyte-subsets mediate hepatic-fibrosis [17] while Natural killer (NK) cells have an anti-fibrotic effect through stimulation of HSCs killing [18]. On the other hand, NK cells are a subset of lymphocytes that play a central role in the innate immune response to tumors and infections [19, 20]. Natural killer (NK) cells are a subset of large granular lymphocytes defined by a lack of T-cell receptor (CD3) and by the surface expression of CD56 [21]. This subset of lymphocytes plays an integral role in the control of a number of viral infections and in tumor cell clearance [22]. Lysosomal-associated membrane protein-1 (CD107a) has been described as a marker of CD8+ T cell and NK cell degranulation following stimulation [20].

Recent studies have found that thyroid hormones, T3 and T4, are important for activation of primary HSCs both in vitro and in vivo [23]. Moreover, hypothyroidism prevents liver cirrhosis in a thioacetamide (TAA) rat model of fibrosis [24] and that hypothyroidism also helps regression of established liver cirrhosis [25].

Because HSCs activation is essential process in the liver fibrosis progressions, it is important to better understand the mechanism(s) by which thyroid papillary carcinoma regulates this process.

Vascular endothelial growth factor (VEGF or VEGF-A) also known as vascular permeability factor (VPF), is a potent mediator of both angiogenesis and vasculogenesis in the fetus and in adults [26]. Its expression correlates with tumor aggressiveness and metastatic potential. An increased expression of VEGF-D and its specific receptors VEGFR-2 and VEGFR-3 has been found in metastatic tissue of papillary thyroid cancer (PTC) and is correlated with an increased lymph vessel density [27, 28]. The data about the serum VEGF levels in subjects affected by thyroid papillary cancer are few [29].

The aim of this study is to investigate the immune-modulatory interactions between thyroid papillary carcinoma and hepatic fibrosis.

## Materials and Methods

### In-vivo studies

#### Animals

Nude-nu male mice (9–10 weeks old) received care accordance with the recommendations in the Guide for the Care and Use of Laboratory Animals of the National Institutes of Health.

The protocol was approved by the Committee on the Ethics of Animal Experiments of the Hebrew University of Jerusalem. All surgery was performed under Ketamine/ Xylazine anesthesia, and all efforts were made to minimize suffering.

#### Mice fibrosis models

Carbon tetrachloride (CCl_4_; Sigma, Cream Ridge, NJ, USA, C-5331) was used to induce hepatic- fibrosis [19, 30]. CCl_4_ was diluted 1:9 with corn oil and administered by bi-weekly intra peritoneal (IP) injections of 0.5 μl pure CCl_4_/g body weight along 6 weeks.

#### Animal experimental design

To study the in vivo thyroid tumorgenicity outcome in liver fibrosis model; we induced thyroid papillary carcinoma cell line (NPA) through subcutaneous (S.C) injections. Four groups of animals were included: (A) Fibrotic tumor bearing animals. (B) Fibrotic animals without NPA injection (C) Non-fibrotic tumor bearing animals (D) Naïve animals without NPA cell line injections. The conditions of the animals were monitored on a daily basis following tumor inductions. Following ketamine/xylazine anaesthesia, mice were killed by cervical dislocation at weeks 6.

#### Serum alanine aminotransferase (ALT) and thyroxine (T4) levels

Blood samples were collected from the inferior vena cava and ALT was measured using an automated enzymatic assay with the Vistros Chemistry Systems 950 while T4 serum levels were measured by immunoradiometric (IRMA) assay (Dynotest Tg S, Brahms, Berlin, Germany).

#### Serum VEGF Measurements

Serum VEGF measurements were performed in batches using the Quantikine Mouse VEGF sandwich enzyme immunoassay (R&D Systems, Minneapolis, MN) after 1–3 months of storage time at -70°C. A polyclonal antibody specific for mouse VEGF has been pre-coated onto a micro-plate. Standards, Control, and samples are pipetted into the wells and any mouse VEGF present is bound by the immobilized antibody. After washing away any unbound substances, an enzyme-linked polyclonal antibody specific for mouse VEGF is added to the wells. Following a wash to remove any unbound antibody-enzyme reagent, a substrate solution is added to the wells. The enzyme reaction yields a blue product that turns yellow when the Stop Solution is added. The intensity of the color measured is in proportion to the amount of mouse VEGF bound in the initial step. The sample values are then read off the standard curve [31].

#### Alpha smooth muscle actin immunoblot

Immunoblot analysis of α smooth muscle actin (αSMA) in liver extracts was performed with modifications as previously described [32]. Whole liver protein extracts were prepared in liver homogenization buffer (50 mmol/L Tris–HCl [pH 7.6], 0.25% Triton-X 100, 0.15 M NaCl, 10 mM CaCl_2_ and complete mini EDTA-free protease inhibitor cocktail (Roche Diagnostics, Mannheim, Germany)). Next, proteins (30 μg per lane) were resolved on a 10% (wt/vol) SDS-polyacrylamide gel (Novex, Groningen, The Netherlands) under reducing conditions. For immunoblotting, proteins were transferred to a Protran membrane (Schleicher & Schuell, Dassel, Germany). Blots were incubated overnight at 4°C in a blocking buffer containing 5% skim milk and then incubated with either anti-αSMA (DAKO, cat no. M0851) or β-actin (Sigma) mouse monoclonal antibody, diluted 1/2000, for 2 h at room temperature, and subsequently, with peroxidase- conjugated goat anti-mouse IgG (PARIS, Compiegne, France) diluted 1/10,000, for 1 h at room temperature. Immunoreactivity was revealed by enhanced chemiluminescence using an ECL kit (Amersham Pharmacia Biotech, Les Ulis, France).

#### Tissue RNA extraction

Total cellular RNA was extracted from target tissues using Trizol reagent (Gibco-BRL, Life Technologies, Grand Island, New York, USA) and followed by DNase I digestion. Purified RNA was then used as a template for reverse transcription into single-stranded cDNA using a reverse transcription system (Promega, Madison, Wisconsin, USA). Synthesized β-actin and αSMA were detected by real-time PCR with the following mice primers: β-actin- (as a housekeeping gene) Forward, 59-GATGAG- ATT-GGC-ATG-GCT-TT-39; β-actin-Reverse, 59-AGA-GAA-GTG-GGG-TGG-CTT-TT-39. αSMA-Forward, 59-TCC-TCCCTG-GAG-AAG AGC-TAC-39; αSMA- Reverse, 59-TAT-AGG-TGG-TTT-CGT-GGA-TGC-39 [30].

#### Real-time PCR analysis

Liver RNA was extracted as described above. Synthesis of cDNA was performed using 2 μg of total RNA per sample with random primers and reagents contained in the Reverse Transcription System kit, according to the manufacturer’s protocol (Promega Corporation, Madison, WI). The reverse transcriptase product was diluted 20 x in nuclease-free H_2_O and 5 μl of each sample was loaded into 96 well plates for real-time PCR in an ABI Prism 7700 Sequence Detection System (Applied Biosystems). β-actin and α2-macroglobulin served as internal controls and H_2_O served as a negative control. Amplification reactions included oligonucleotide primers for each target gene, and for β-actin and α2-macroglobulin, as well as platinum Taq polymerase and SYBR Green DNA-binding dye. Fluorescence signals were analyzed during each of 40 cycles (denaturation 15 s at 95°C, annealing 15 s at 56°C and extension 40 s at 72°C). [Denaturation curves of target genes and β-actin, performed at the end of the PCR], and detection of the PCR products by agarose gel electrophoresis confirmed the homogeneity of the DNA products. Relative quantification was calculated using the comparative threshold cycle (C_T_) method [as described in the User Bulletin #2, ABI PRISM 7700 Sequence Detection System]. C_T_ indicates the fractional cycle number at which the amount of amplified target genes a fixed threshold within the linear phase of gene amplification, and is inversely related to the abundance of mRNA transcripts in the initial sample. Mean C_T_ of duplicate measurements would be used to calculate ΔC_T_ as the difference in C_T_ for target and reference. ΔC_T_ for each sample was compared to the corresponding control C_T_ and expressed as ΔC_T_. Relative quantity of product was expressed as fold-induction or repression of the target gene compared to the control primers, according to the formula 2^-ΔCT^ [19].

#### Liver /Spleen lymphocyte and NK isolation

Intra-hepatic and splenic lymphocytes were isolated by perfusion of the liver with digestion buffer. After perfusion, the liver was homogenized and incubated at 37°C for 30 min. The digested liver/spleen cell suspension was centrifuged to remove hepatocytes and cell clumps. The supernatant was then centrifuged to obtain a pellet of cells depleted of hepatocytes to a final volume of 1 ml. Lymphocytes were then isolated from this cell suspension using 24% metrizamide gradient separation [31]. Cells were cultured or counted and stained for FACS analysis. For spleen cells; the spleens were meshed through cell strainers (40μm BD FALCON, USA), cells were sedimented by centrifugation at 800xg for 3 minutes, and then treated with lysis buffer at RT for 3 minutes, 9mL DMEM added and spinned as before [33]. Liver/spleen NK from lymphocytes were further isolated using a magnetic cell sorting kit (Miltenyi Biotec) according to manufacturer’s instructions.

#### Histological assessments of liver injury

The posterior one third of the liver fixed in 10% formalin was paraffin-embedded in an automated tissue processor. Seven-millimeter liver sections were cut from each animal. Sections (15mm) were then stained for Hematoxylin and eosin (H&E) staining for each animal. Knodell score was assessed blindly by an expert hepatic pathologist (M.I.F.) based on H&E, using the modified Histological Activity Index (HAI) criteria, incorporating semi-quantitative assessment of periportal/periseptal interface hepatitis (0–4), confluent necrosis (0–6), focal lytic necrosis/apoptosis and focal inflammation (0–4) and portal inflammation (0–4).

#### Fluorescence-activated cell sorting (FACS) analysis

Harvested NK cells were adjusted to 10^6^/ml in staining buffer (in saline containing 1% bovine albumin) and were incubated with antibody on ice for 30 min, washed with staining buffer and fixed with 2% paraformaldehyde. Fc receptors were blocked by incubation with 1% human plasma for 15 min on ice. Lysosomal-associated membrane protein-1 (CD107a) as a surface staining has been described as a marker of CD8+ T cell and NK cell degranulation following stimulation [34]. CD107a is significantly up-regulated on the surface of NK cells following stimulation and was therefore used in our study.

### In-vitro studies

#### Thyroid cancer cell line

The human thyroid cancer cell line NPA was used as papillary thyroid carcinoma cell line [35, 36]. These cell lines were cultured according to the recommendations of the cell bank. The cell lines were maintained in DMEM supplemented with 10% fetal bovine serum (FBS) and penicillin (100 U/mL)-streptomycin (100 g/mL) at 37°C in a humidified 5% CO2 atmosphere.

#### Co-culture conditions

To study the direct interaction between NK cells and NPA cell line, NK cells derived from the 4 animal groups were co-cultured with NPA in 18 mm dishes (Nunc Brand Products, Roskilde, Denmark) 10% FCS (Atlantic Biologicals) and incubated for 48h—6 days at 37°C. Adhered- NPA cells were trypsinized/washed for analysis for NPA proliferations by CFSE using flow-cytometry (As described below).

#### Proliferation Assay

NPA cells were washed and resuspended with PBS, and incubated for 10 min at 37°C with CFSE (5,6 carboxyfluorescein diacetate succinimidy ester, which was purchased from Invitrogen,uregon, USA) at a final concentration of 1μmol/ml). Briefly, CFSE staining of NPA cells after labeling is extremely high fluorescence. The majority of CFSE initially taken up by the cells is lost within the first few days following proliferation. The more the decrease in fluorescence the higher the cells proliferate. The units obtained are the Mean fluorescence intensity (MFI- arbitrary unit). CSFE-proliferations changes in day 3 and day 5 were compared to day 0 of CSFE staining. Proliferations fold changes analyzed by flow cytometry were calculated by divided day 0 to day 5.

#### Statistical Methods

Results are presented as mean values ± standard deviation. Student’s T-Test, Mann-Whitney, Krruskat-Wallis and ANOVA were used to evaluate statistical significant correlations between study groups.

## Results

### Increased in papillary thyroid tumorgenicity following inductions of CCl_4_-hepatic fibrosis

**Fig 1:**
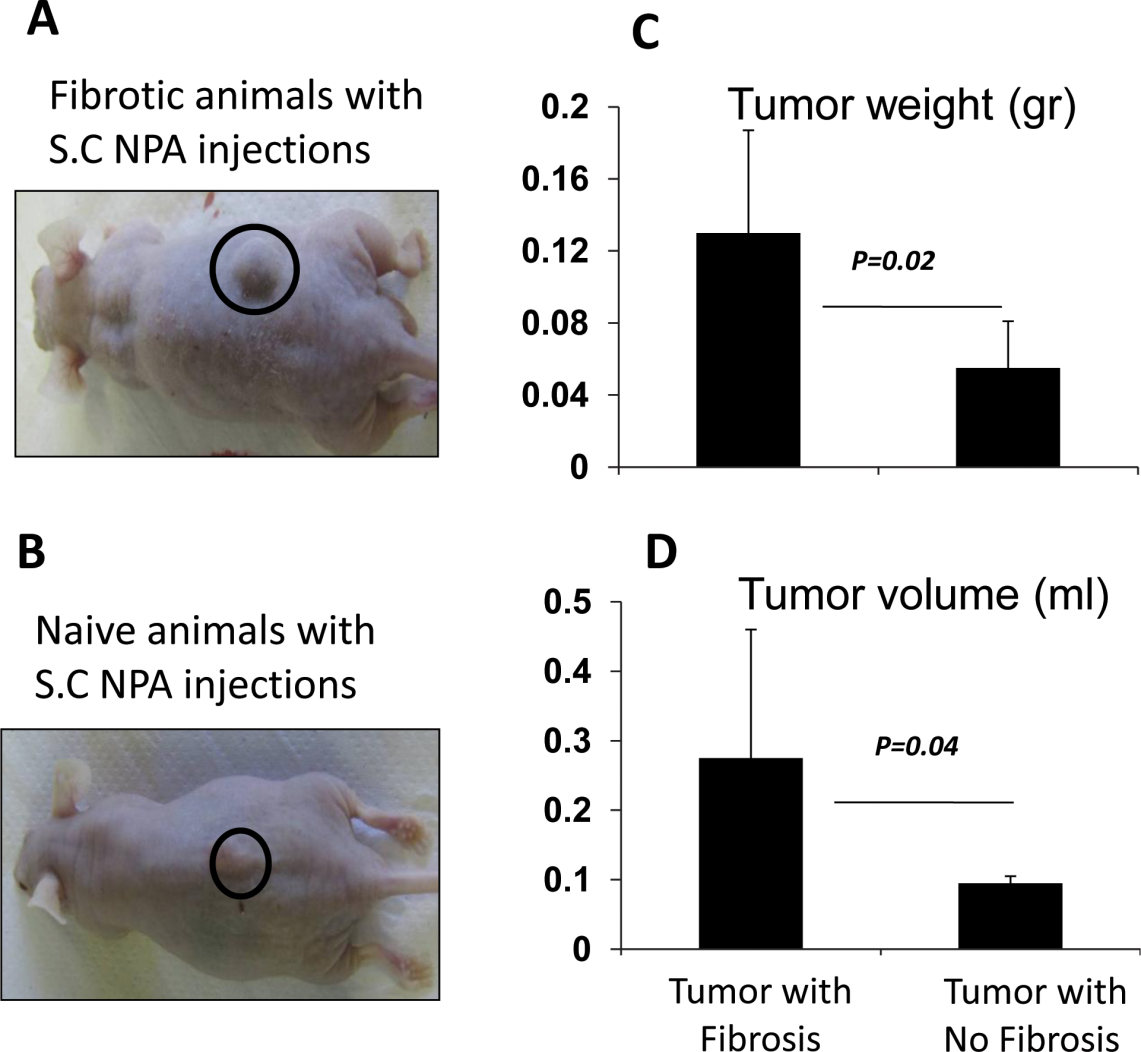
Hepatic fibrosis increase NPA tumor weight and size. In vivo S.C injection of NPA cells model was performed as described in M&M. In this model; S.C NPA-cells tumor was induced in fibrotic and naïve (no fibrosis) animals. S.C tumors were explanted at the end of 6 weeks post S.C injection, and evaluated for tumor weight and volume. (A) and (B) show the external appearance of the tumor in the animal’s back. Tumor weight (C) and volume (D) was increased significantly from (0.13±0.06gr) and (0.28±0.18ml) in the fibrotic group to (0.05±0.025 gr) and (0.09±0.01ml) in the non-fibrotic group; p-value = 0.02 and 0.04, respectively.

In order to determine the tumorgenicity outcome of thyroid papillary carcinoma in liver fibrosis model; *in vivo* S.C injection of NPA cells was performed as described in M&M. In this model; S.C NPA tumor cells was inoculated in fibrotic model receiving the CCl_4_ and in naïve (no fibrosis) animals. Fibrotic and naïve animals groups without NPA tumor induction were included as a control groups. S.C tumors were explanted at the end of 6 weeks post S.C injection, and evaluated for tumor weight and volume (Fig 1C and 1D). The macroscopic examination of the tumors in the area of inoculation in Fig 1A and 1B is showing a bigger tumor growth in the back of the fibrotic animals as compared to the naïve ones (non-fibrotic group) with the tumor inoculation. Furthermore, our data showed that tumor weight and volume was increased significantly from (0.13±0.06gr) and (0.28±0.18ml) in the fibrotic group to (0.05±0.025 gr) and (0.09±0.01ml) in the non-fibrotic group; p-value = 0.02 and 0.04, respectively (Fig 1C and 1D). The data suggests a prominent tumoral effect in hepatic fibrosis model.

### Papillary thyroid tumor increases severity of hepatic fibrosis and liver injury

**Fig 2:**
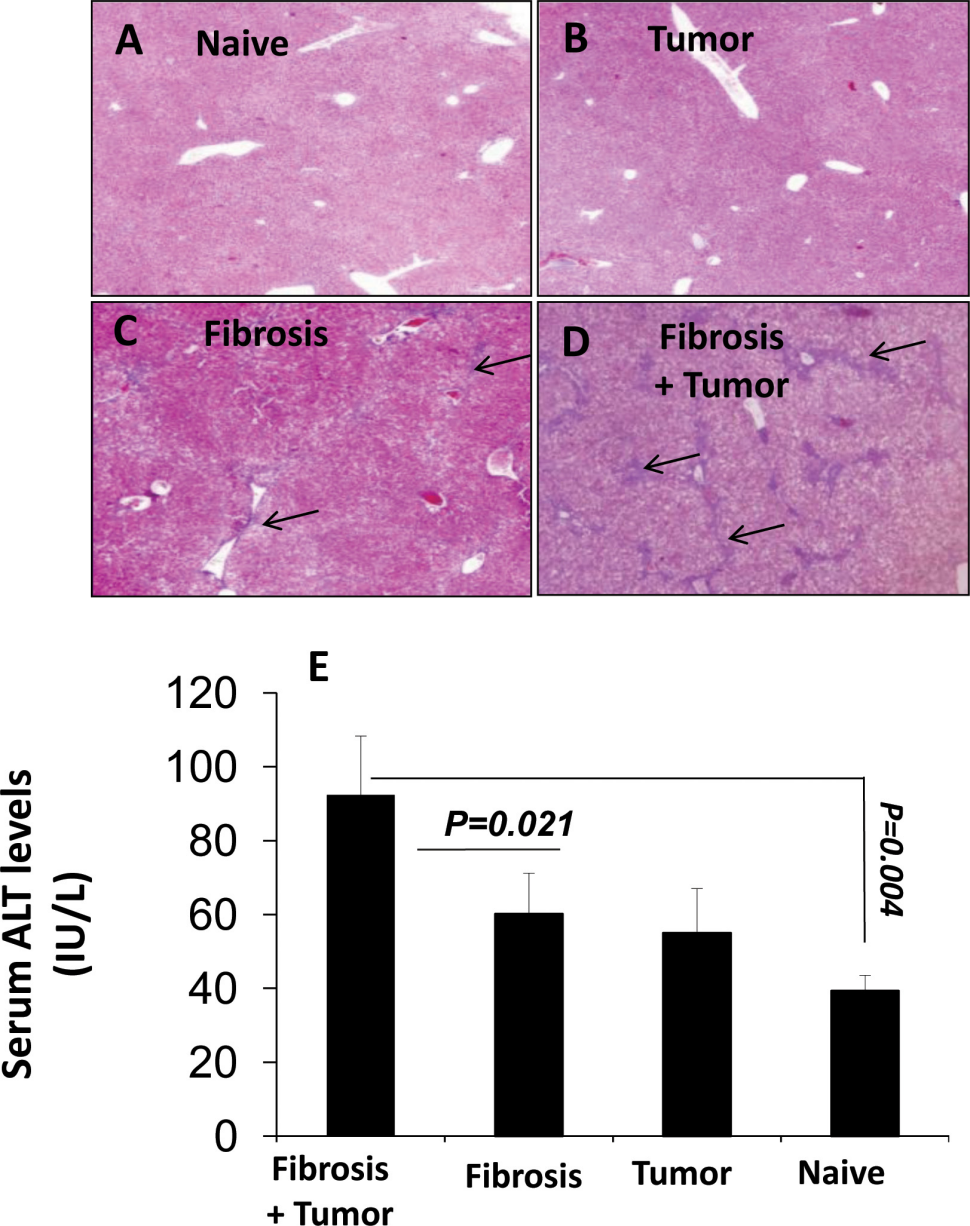
NPA Tumor inductions increase the severity of CCl4 related hepatic injury. CCl4-hepatic injury was evaluated by hematoxylin and eosin (H&E) staining of necro-inflammatory liver lesions and ALT serum levels. Immunohistochemical staining with H&E (5X magnification) for the four major animal groups showed necro-inflammatory lesions and cell infiltrations that were increased in the fibrotic mice receiving the NPA-tumor cells (D) as compared to fibrotic alone (C). Arrows indicate the area with lymphocyte infiltrations. No inflammatory infiltrates were seen in H&E staining of (A) naïve WT and (B) naïve mice receiving the NPA-tumor cells. (E) Serum ALT levels were in line with histological findings and showed increase from (60±25/L) in fibrotic animals without tumor to (85.5 ± 20.5 U/L) in animals with tumor and hepatic fibrosis; p-value = 0.021.

CCl_4_-hepatic injury was evaluated by immunohistochemical staining with H&E of the necro-inflammatory liver lesions for the animal groups with the NPA inoculation as well as with serum ALT levels and by (Fig 2). Following the S.C NPA-injections, secreted serum ALT levels were weekly obtained. The results obtained following 6 weeks of NPA tumor inoculation showed that H&E staining showed necrotic infiltrations that were increased in the fibrotic mice receiving the NPA cells (Fig 2D) as compared to the fibrotic mice without the NPA transplantations (Fig 2C). No inflammatory infiltrates were seen in H&E staining of naïve WT with (Fig 2A) or without the tumor (Fig 2B).

ALT serum levels were significantly increased in fibrotic tumor bearing animals (92.3 ± 18.7 U/L) as compared to fibrotic animals without tumor (66±14U/L; p-value = 0.021, Fig 2E). Both the naive animals (no tumor and no liver fibrosis) and the non-fibrotic tumor bearing ones had similar levels of reduced serum ALT (p-values were statistically not significant)

The ALT results were in line with liver histology and indicate that NPA tumor induction to fibrotic animals increase severity of liver injury as compared to the naïve group or to the non-tumor fibrotic group.

**Fig 3:**
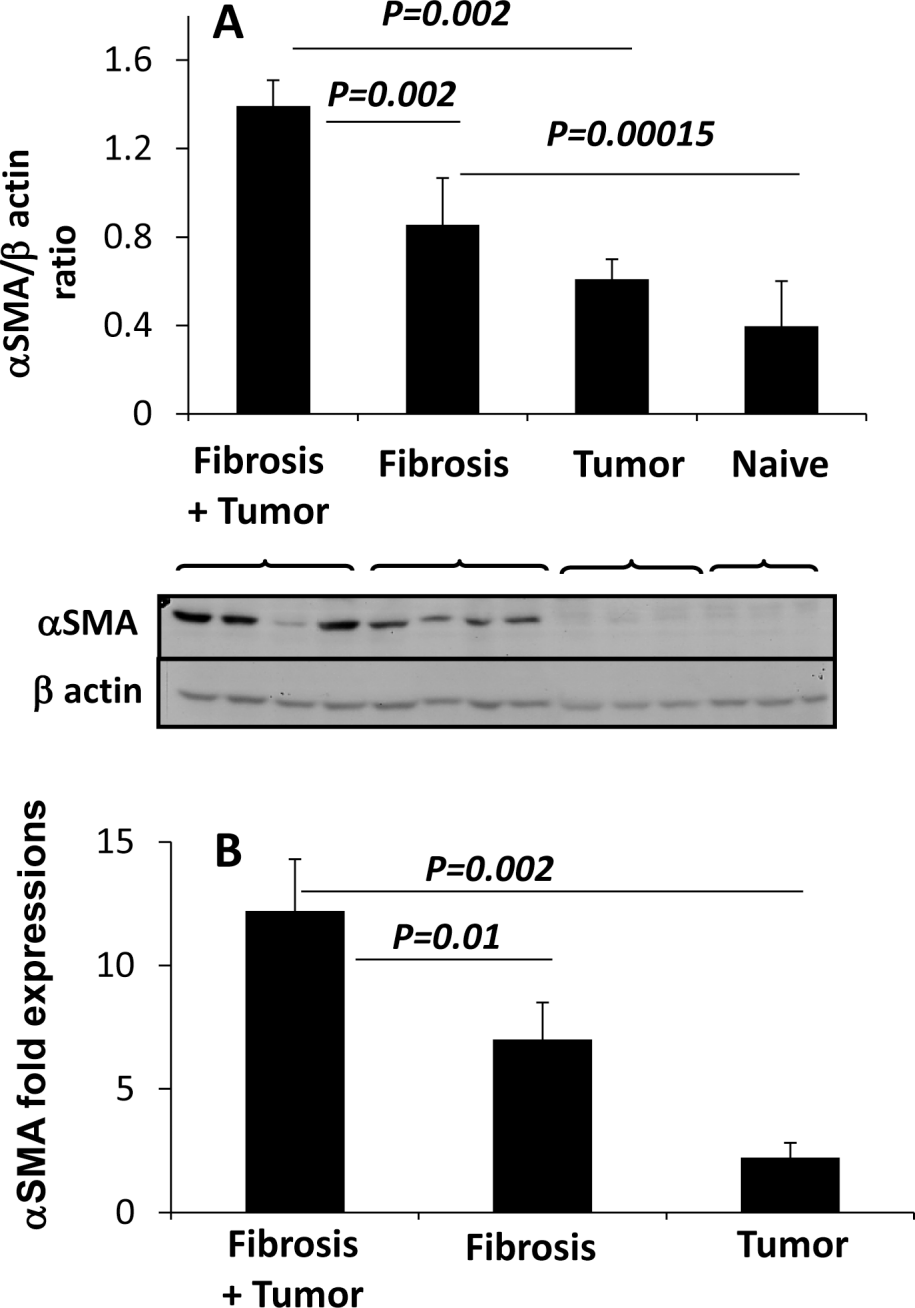
Tumor increase severity of hepatic fibrosis. Fibrotic profile was estimated by (A) Western blot quantitations and (B) RT-PCR expressions of α smooth muscle actin (αSMA). (A) (Lower panel) displays a representative membrane with examples of αSMA expression as a marker for the HSCs activation (upper bands) and GAPDH (lower bands) expression by western blotting in the harvested liver protein extracts. (A) (Upper panel) shows the calculated ratio of αSMA/β-actin based on the densitometry readings of the bands. (B) Real-time PCR data reflect changes in gene expression of αSMA mRNA expressed as fold change compared with naïve mice. Expression of αSMA mRNA corresponded to the western blot results. Experiments were repeated 4 times, in each time 4 mice were included in each group. Averages of the 4 experiments were included in the quantitations of western blots and RT PCR.

We next investigated fibrotic profile by quantifying the expressions of HSCs activation marker; alpha smooth muscle actin (αSMA), using western-blots and RT-PCR (Fig 3). Using western blotting from animals’ liver extracts, our results showed prominent expressions of αSMA in the fibrotic animals receiving the NPA cells as compared to the NPA-untreated fibrotic mice (Fig 3A, lower panel). Fig 3A (upper panel) shows the calculated ratio of αSMA/β-actin based on the densitometry readings of the bands. The measured αSMA/ β-actin ratio was significantly increased to (1.4±0.1) in fibrotic animals receiving the NPA cells when compared to NPA-untreated fibrotic mice (0.8±0.2; p-value = 0.002). Both naïve NPA-untreated group (no tumor and no fibrosis) and NPA-treated group showed low expressions of αSMA.

Expressions of mRNA of the αSMA (Fig 3B) were in line to the western blot results. Real-time PCR data reflect changes in gene expression of αSMA mRNA expressed as fold change compared with naïve mice. Data is showing 12.7±1.6-fold increase in αSMA mRNA expression on liver tissue from fibrotic animals receiving the NPA cells as compared to 7.0±1.1-fold in NPA-untreated fibrotic mice and 2.2±0.3 in naïve with the tumor (Results are highly significant).

As correlated with our previous studies [37], and as we demonstrate in our current data; hepatic fibrosis was significantly increase in the CCl_4_ fibrotic group compared the non-fibrotic group. Moreover, these results showed a significant increase of hepatic fibrosis following NPA cell induction in fibrotic mice compared with the non-tumor group.

### NK cells lost their anti-fibrotic potential in the fibrosis-bearing tumor mice

**Fig 4:**
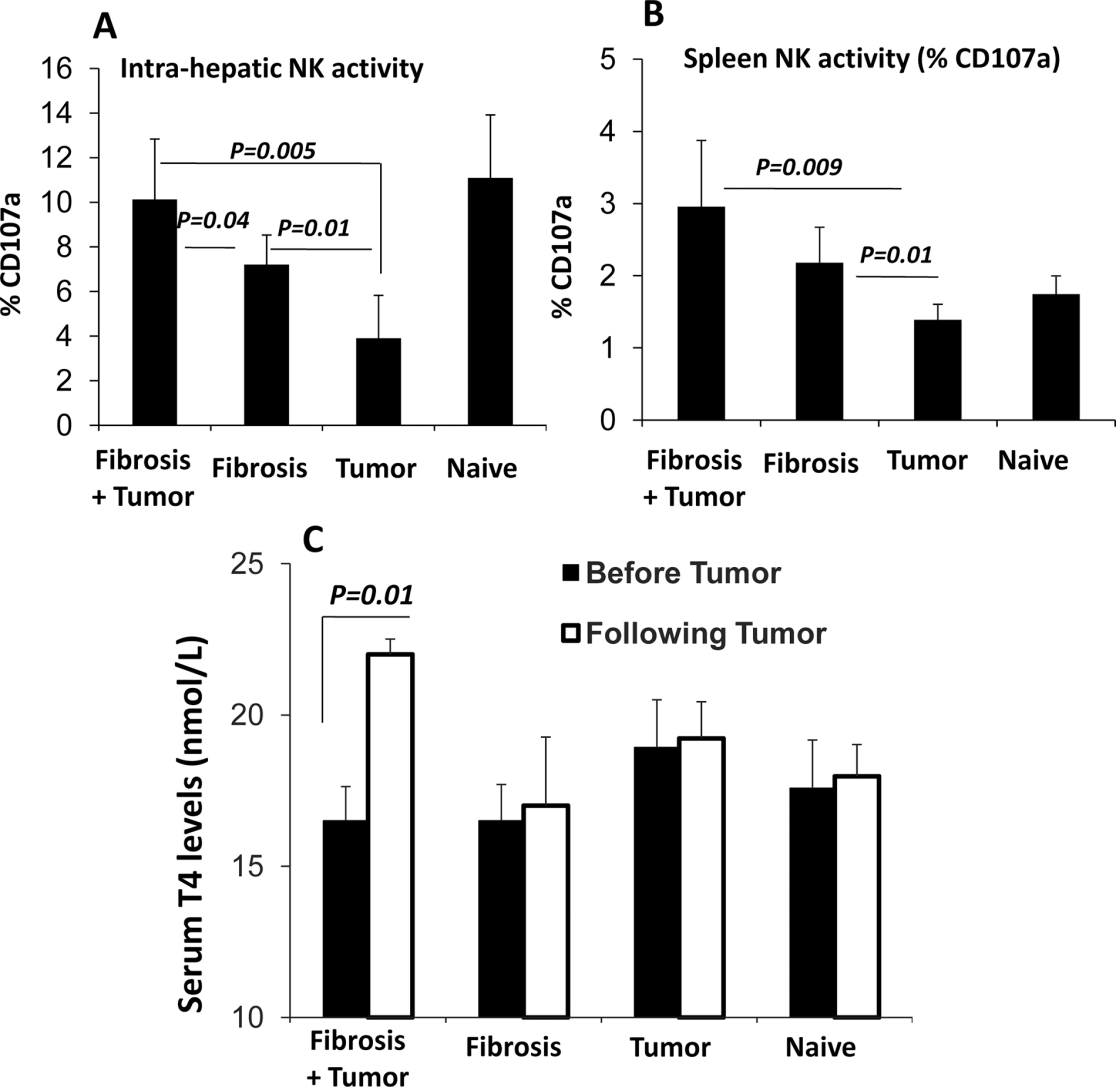
NK cell activity and T4 excretions increased in the fibrotic group with NPA tumor induction. (A) Intra-hepatic NK cells as well as (B) NK from spleen from four groups were isolated and stained for NK activity (CD107a) using flow-cytometry. The data showed a significant activation of the intrahepatic and splenic NK from the fibrotic group with NPA tumor induction compared to the non-tumor injected animals. (C) Shows serum T4 levels significantly elevated in animal models of fibrosis following NPA tumor injections.

We have previously showed that NK cells have anti-fibrotic effects through killing of activated HSCs [33]. We next investigated whether NK cells could still preserve their anti-fibrotic potentials in our fibrotic model after induction of the NPA tumor cells. CD107a (lysosomal-associated membrane protein-1) is significantly up-regulated on the surface of NK cells following stimulation and was therefore used in our study as a marker of NK activity. Intra-hepatic NK cells as well as splenocytes were isolated from the four studied mice groups and were evaluated for NK activity (CD107a) using flow cytometer, as previously described. Fig 4 shows high expressions of CD107a on NK cells from fibrotic animals with NPA tumor. The liver NK cells expressions of CD107a increased from 7.21±1.3% in fibrotic animal to 10.13 ±2.7% in the fibrotic tumor bearing animals; p-value <0.05. In addition, CD107a expressions were significantly decreased to 3.9 ± 1.9% in the non-fibrotic tumor bearing animals; p-value <0.01. Similar patterns were seen in the splenic NK; Fig 4B. The data showed a significant activation of the intrahepatic and splenic NK from the fibrotic group with NPA tumor induction compared to the non-tumor animals.

Recent studies have found that thyroid hormones such as T4 activate HSCs [22]. We aimed to assess serum T4 levels in our fibrotic models with or without tumor inductions. Blood samples were collected from the retro-orbital sinus prior to tumor induction and at the endpoint time. Serum T4 levels were elevated in the fibrotic and naïve group after NPA tumor injection (Fig 4C). In the fibrotic tumor bearing mice, serum T4 level was increased from 16±2.2-nmol/L pre-tumor inoculation to 19.6±0.5 nmol/L after tumor induction; p-value = 0.01. In the non-fibrotic tumor bearing mice, serum T4 level was unchanged post tumor induction (Fig 4C). No significant results were obtained from the naïve and fibrotic groups. Our result demonstrates that Serum T4 levels were significantly elevated in fibrosis animals after NPA tumor injection.

The current results showed in spite of increased NK cell activity, the NKs lost their anti-fibrotic potentials to inhibit hepatic fibrosis probably due to high serum T4-excretions.These results could be consistent with other data [22] which emphasize the role of thyroid hormones in liver fibrosis.

### Increased NK cell activity is suggested to have anti-proliferative tumor cell effects

**Fig 5:**
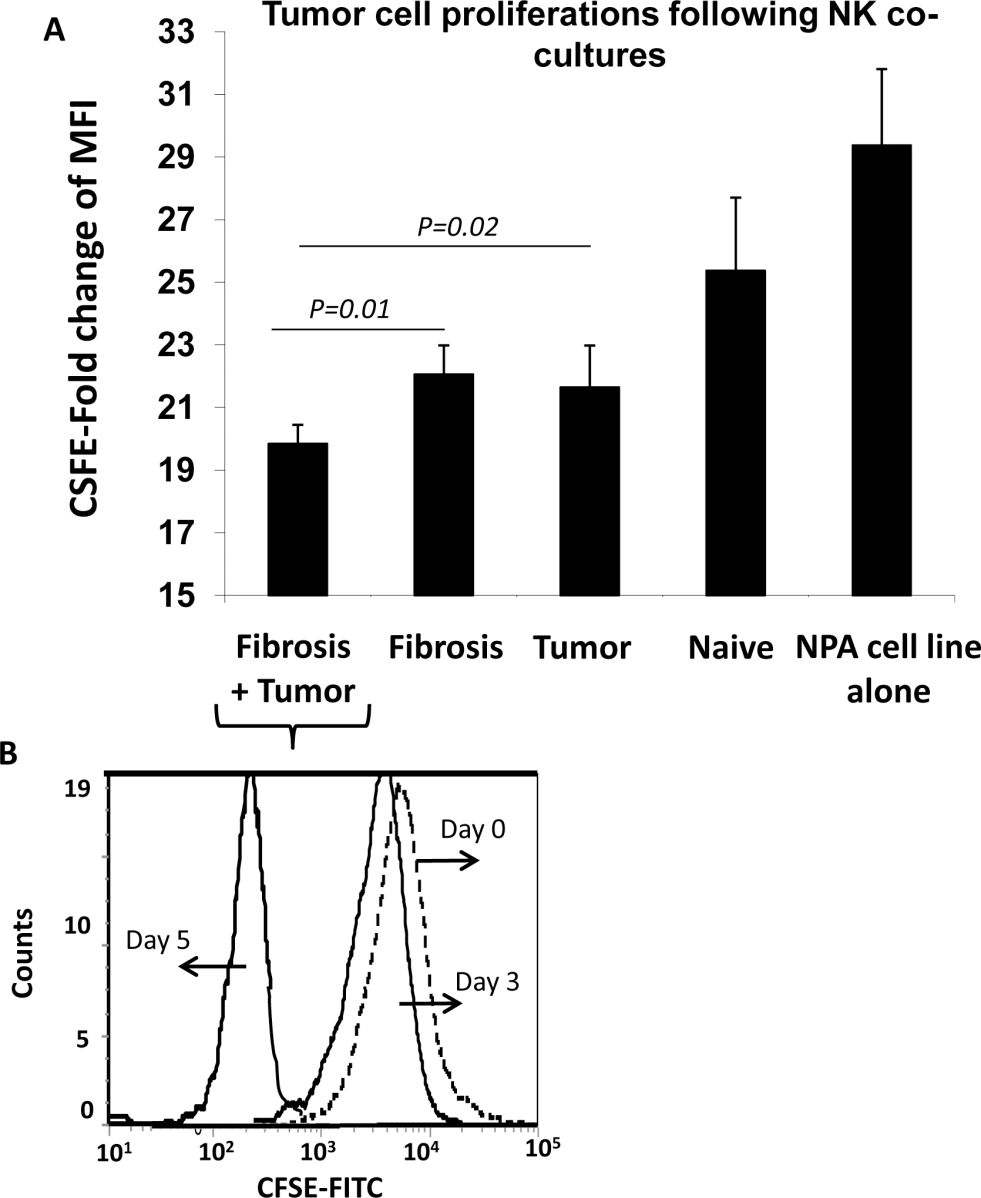
In vitro co-culture of lymphocytes with NPA cell line. Adhered-NPA cells post co-culture with NK cells from different animal groups were analyzed for proliferation by CFSE using flow cytometry. (A) Direct co-culture of NPA cells with spleen NKs from fibrotic mice with tumor significantly decreased NPA tumor cell proliferation compared to the fibrotic mice without tumor, indicating highly stimulated NK cells effects; p-value = 0.001. (B) A representative histogram of the NPA cells following incubations with NK cells of fibrosis and tumor mice. The histogram shows CSFE-proliferations changes in day 3 and day 5 as compared to day 0 of CSFE staining. Proliferations fold changes were calculated by divided day 0 to day 5.

In order to study the direct effects of NPA cell line on immune lymphocyte alterations; splenocytes derived from the different animal groups were in-vitro co-cultured with NPA tumor cells. Adhered- NPA cells post co-cultures with lymphocytes were analyzed for proliferation by CFSE using flow cytometry as described in M&M. In Fig 5A, NK cells obtained from fibrotic mice with tumors caused decreased in NPA tumor cell proliferations to 19.85±0.6-fold as compared to their counterparts obtained from the naïve group. In additions, NK cells from the fibrotic as well as the tumor animals induced decreased levels of NPA proliferations to 22.05±0.9-fold and 21.7±1.3-fold; respectively. Fig 5B shows a representative histogram of the NPA cells following incubations with NK cells of fibrosis and tumor mice. The histogram shows CSFE-proliferations changes in day 3 and day 5 as compared to day 0 of CSFE staining. The most significant changes were seen following day 5, therefore proliferations fold changes were calculated by divided day 0 to day 5.

Among all four groups, NPA tumor cell proliferation level was the lowest when co-culture with NK cells form the fibrotic tumor bearing mice and as showed earlier in this study, this group had elevated expressions CD107a indicating an increased in their NK cells activities. All together, our data indicated that increased NK cell activity is suggested to have anti-proliferative tumor cell effects, and on the other hand; in the in-vivo setting, fibrosis increase tumor mass in spite of increased NK activity. Although NK cells play a central role in the innate immune response to tumors [20], this anti-tumor effect in NPA tumor development was not sufficient to keep better outcome of tumor growth in spite of increased NK cell activation, probably due to pro-tumor serum mediators.

### Increased VEGF serum levels in the fibrosis-bearing tumor mice

**Fig 6:**
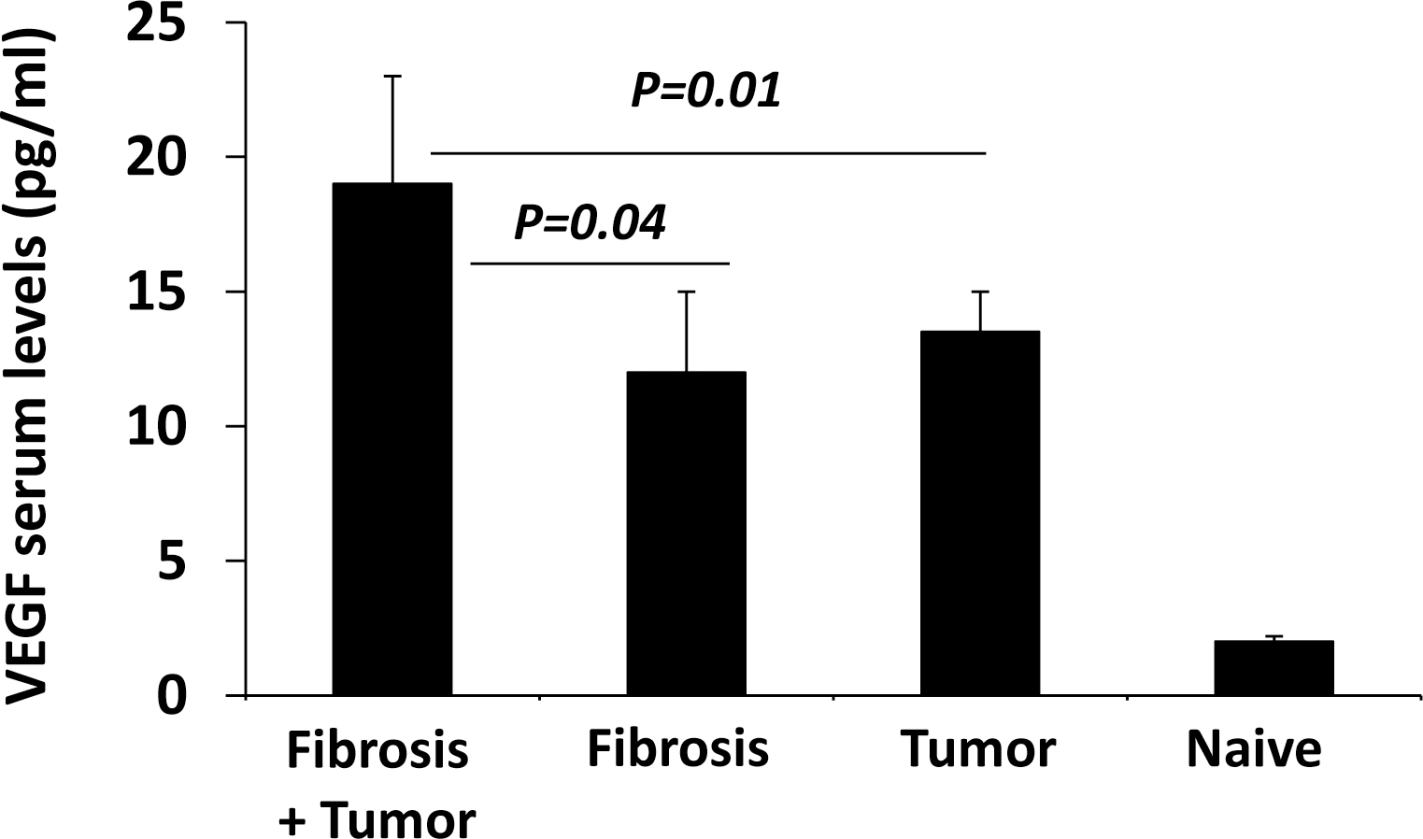
VEGF serum levels. Quantikine Mouse VEGF immunoassay serum levels was significantly increased in the fibrotic group bearing tumor as compared to tumor alone (p = 0.01) or fibrosis alone groups (p = 0.04). No statistical significant differences were found in VEGF serum levels between the fibrotic and non-fibrotic group with tumor induction.

Taking all the data together, we next sought to determine potential effects of pro-tumor serum mediators which could play an important role for thyroid tumor development; we used the Quantikine Mouse VEGF immunoassay and the IRMA assay in order to detect the serum level of vascular endothelial growth factor (VEGF) and Estradiol, respectively. Estradiol serum levels were below the detected ranges of the IRMA assay (data not shown). Fig 6 showed that VEGF serum levels at the end point (euthanasia) were significantly increased from 1.5±2.2 pg/ml in the naïve group to 12.1±6.2 pg/ml and 13.7±2.3 pg/ml in the fibrotic group without tumor induction and in the non-fibrotic tumor bearing group, respectively. Statistical significant of elevated VEGF serum levels were found in the fibrosis-bearing tumor mice as compared to both the fibrotic and non-fibrotic tumor-bearing group with tumor induction.

## Discussion

In the present study we investigated the effects of liver fibrosis in the progressions of NPA papillary thyroid carcinoma. In order to address the study objectives, we applied in vitro and in vivo setting studies. In the vivo settings, nude mice were induced for liver fibrosis by CCl_4_ injections and were then tested for thyroid tumorgenicity through subcutaneous administrations of NPA papillary thyroid carcinoma. In the in-vitro settings, co-culture conditions representing the interactions between NPA cell line and lymphocytes derived from the different animal groups.

Chronic liver injury caused by viral hepatitis, alcohol and drugs results at first in fibrosis, and if the injury persists, leads to liver cirrhosis [15]. The incidence of thyroid cancer has more than doubled in recent decades [38–40]. Debate continues on whether the increasing incidence is a result of an increased detection of small neoplasms or other factors. Recently the data indicate that the increasing incidence of thyroid cancer cannot be accounted for fully by an increased detection of small neoplasms [41]. Antonelli et al. showed a significantly higher prevalence of papillary thyroid cancer in HCV+ patients than in naive controls [41, 42]. The precise mechanism by which HCV causes these malignancies is unknown. Moreover, an oncogenic role for HCV has been demonstrated in the case of hepatocellular carcinoma complicating chronic HCV infection, with or without cirrhosis as an intermediate [43]. Because HCV is an RNA virus that cannot be integrated in the host genome, its oncogenic potential must be exerted through indirect mechanisms [44, 45]. Therefore, the results of the epidemiologic studies showing an association between HCV infection and thyroid cancer need to be confirmed; what mechanisms transduce the HCV oncogenic. Non–liver-related malignancies are increasingly seen in patients infected with hepatitis C virus (HCV). Whether this phenomenon is related to the increasing prevalence of chronic hepatitis C (CHC) or to a direct causal role by HCV is unknown [46].

Natural killer (NK) cells are a key component of the innate immune system and play a critical role in the early stages of the immune response against tumor cells, as well as those infected by viral and microbial pathogens [47]. NK are immune cells sensing and eliminating foreign, stressed, transformed and senescent cells through specialized surface receptors [48]. On the other hand, our previously results imply that NK cells are an anti-fibrotic subset, a conclusion supported by a number of clinical observations in chronic liver diseases [19].

In the current study, we showed that NPA tumor cell line caused a significant increase in NK activity in CCl_4_ hepatic fibrosis model. Fig 4A and 4B showed a significant activation of the intrahepatic and splenic NK from the fibrotic tumor bearing group compared to the non-tumor injected animals. In addition, NPA tumor induction increases serum T4 secretions in the circulation. Blood samples were collected from the retro-orbital sinus prior to tumor induction and at the endpoint time. Serum T4 levels were unchanged in the fibrotic and naïve tumor bearing groups after tumor injection while our data demonstrated significantly elevated serum T4 levels after NPA tumor injections in the fibrotic animals (Fig 4C). Furthermore, these results showed a significant increase of hepatic fibrosis after NPA cell induction in fibrotic group with NPA tumor compared the non-tumor fibrotic group (Fig 2). NPA tumor induction to fibrotic animals increased significantly the severity of liver injury (evaluated by serum ALT levels and H&E- stained necro-inflammatory liver lesions) compared to the naïve group or to the non-tumor fibrotic group. Fibrotic profile estimated by HSCs αSMA expressions and quantitations showed also increased in hepatic fibrosis after NPA cell induction compared the non-tumor group.

In our experimental model, although NK cells have an anti-fibrotic effect in liver fibrosis [19], CCl_4_-injected mice with tumor induction still develop increased fibrosis, in spite of the higher state of NK activation, suggested that the anti-fibrotic effect of NK cells was not sufficient enough to decrease fibrosis in our experiments, probably due to high serum T4-excretions (Fig 4) [22]. In addition, these results might confirm others [22], which emphasize that thyroid hormones play an important role in activation of primary HSCs and liver fibrosis. The main effect of T3 and T4 is up-regulation of αSMA expression and accelerated activation of HSCs in vitro, and the effect of T3 on αSMA is transcriptional and abolished by T3 antagonist [22]. Recent studies have found that thyroid hormones, T3 and T4, are important for activation of primary HSCs both in vitro and in vivo [22]. They show that T3 and T4 enhance activation of HSCs by three different mechanisms: direct up-regulation of αSMA, increased expression of p75NTR and, rapid direct activation of Rho [22].

Our data also suggested a prominent tumoral effect of hepatic fibrosis in NPA tumor model. Tumor weight and volume was significantly increased in the fibrotic group compared to the non-fibrotic group (Fig 1) in spite of increased NK activation. In the in vitro setting, NPA tumor cell co-cultured with the expected activated NK cells derived the fibrotic tumor bearing mice significantly decreased their proliferation compared to the fibrotic mice without tumor (Fig 5).

In the current study, we propose that NK cell alterations in their release of granzymes (CD107a) are involved in the tumorgenicity of thyroid papillary carcinoma. Although NK cells play a central role in the innate immune response to tumors [17]; fibrotic animals with sustained NK activity still propagate tumor mass, in spite of increase NK activation, suggesting that anti-tumor effect of NK cells in NPA tumor development was not enough sufficient to keep better outcome of tumor, probably due to pro-tumor serum mediators. These results might confirm others, which suggest those pro-tumor serum mediators; including hormones, cellular proteins [2], platelet-activating factor (PAF) [40], serum response factor (SRF) [35]; are important for thyroid tumor development.

An increased expression of VEGF, along with VEGF-C, and their receptors VEGFR-2 and VEGFR-3 are reported in thyroid cancer and correlated with lymph node metastasis [49, 50]. Vascular endothelial growth factor (VEGF), a principal stimulant to endothelial cell growth and migration, is a 32-to 46-kDa homodimeric glycoprotein that promotes endothelial regeneration, stimulates the formation of collateral blood vessels, increases vascular permeability, and inhibits the function of antigen-presenting cells [51]. Several reports suggest that expression of VEGF by thyroid cancer cells is associated with a more aggressive phenotype in both animal models and clinical studies [34].

On the basis of the large body of information demonstrating the importance of VEGF expression in thyroid cancer, we hypothesized that serum VEGF levels would be significantly higher in the mice with the larger mass tumor. Furthermore, because the substantially greater incidence of thyroid cancer in women compared with men and the peak incidence during the reproductive years in women and the fact that hypogonadism are common in patients with liver cirrhosis has led us to hypothesize that estradiol serum level would be increased in the fibrotic tumor bearing mice.

In the current study, the estradiol serum levels were below the detected level of the assay. More studies are warranted to investigate the role of Estradiol in thyroid papillary carcinoma. On the other hand, VEGF level was significantly increased in the fibrotic groups and in the non-fibrotic tumor-bearing group compared to the naïve one. In additions, fibrotic tumor bearing animals showed elevated levels of serum VEGF levels and were significant as compared to both fibrotic and non-fibrotic tumor-bearing groups. These results suggest that the tumorgenicity of thyroid papillary carcinoma in fibrotic mice could be affected by serum levels of VEFG in additions to several factors that are mentioned in literature.

The molecular and immunological mechanisms underlying the pro-malignant actions of HCV are manifold and not completely characterized. Accumulating evidences demonstrated that antiviral therapy can reduce the risk of some malignancies and can increase tumor-free survival following successful tumor-directed therapy. Thus, the role of antiviral therapy for CHC could possibly be expanded to include treatment of certain subgroups of patients with the associated cancers if HCV is identified as a causative agent of these malignancies [49].

In conclusion of the current study results, we demonstrate that NK activation accompanied tumor progression was not effective as anti-tumor or anti-fibrotic. NPA tumor cell increased NK cells activity in CCl_4_ hepatic fibrosis model and increased fibrosis severity, probably due to stimulated effects of serum T4 effect on HSCs activations. On the other hand, hepatic fibrosis increased tumor mass, m/p due to serum pro-tumor mediators. Further immune-molecular and mechanistic investigations of the relationship between HCV/hepatic fibrosis and thyroid carcinoma are warranted. These investigations may contribute to the understanding of the oncogenic process of thyroid papillary carcinoma and to establish a therapeutic approach for treatment of HCV-related thyroid carcinoma.
